# Exercise training alters the glycemic response to carbohydrate and is an important consideration when evaluating dietary carbohydrate intake

**DOI:** 10.1186/s12970-018-0259-2

**Published:** 2018-10-26

**Authors:** Charles Paul Lambert

**Affiliations:** Stautzenberger College, 1796 Indian Wood Circle, Maumee, OH 43537 USA

**Keywords:** Exercise, Insulin, Glycemic response carbohydrate

## Abstract

Carbohydrates raise insulin concentrations in blood. Exercise decreases the insulin response to carbohydrate infusion and is beneficial in reducing postprandial glucose and insulin concentrations. This is important as there has been recent information suggesting postprandial insulin concentrations are linked to obesity (Carbohydrate-Insulin Model of Obesity). The validity of this model may be in question in face of chronic exercise.

## Main text

Recently, the glycemic index of carbohydrate containing foods has received much attention due to the carbohydrate-insulin model of obesity, which has some supporting evidence [1]. The hypothesis of the carbohydrate-insulin model of obesity is that a high glycemic index carbohydrate intake will increase insulin concentrations and therefore increase body fat deposition. Whether or not this is true is still a matter of scientific inquiry. Clearly, an important intervention to reduce the glycemic index of carbohydrate containing foods and greatly reduce the deleterious effects of the carbohydrate-insulin model of obesity is physical exercise [2]. King et al. [2] reported that when the plasma glucose was raised to approximately 450 mg/dl by glucose infusion there was a 64% reduction in insulin secretion in aerobically exercise trained men than in non-aerobically exercise trained men. Interestingly, only 1 week of aerobic exercise training **i**n non-insulin dependent diabetics resulted in a 32% reduction in the area under the insulin curve in response to 100 g of glucose [3]. With regard to glucose disposal, Vukovich et al. [4], showed that endurance runners who quit running for 6 days has a 29% decrease in glucose disposal, suggesting that regular exercise training has a profound effect on increasing glucose disposal. Kirwan et al. [5] reported that 7 days of vigorous exercise induced significant improvements in glucose disposal in type II diabetics. Arciero et al. [6] found that 10 days of aerobic exercise (~ 50–60 min/day) was 37% more effective than a 10 day low calorie diet (50% of initial energy needs, 50% CHO, 35% Pro, 15% Fat) on improving glucose disposal during a hyperglycemic clamp. Although not reported, the energy balance in each group would have been about the same or ~ 300–400 kcal more positive in the exercise group. The mechanism of action for the effect of exercise on glucose disposal is likely the reduction in muscle glycogen with prolonged exercise (~ 50–60 min/day) which is supported by Nolte et al. [7] who reported that epinephrine induced glycogen depletion resulted in major improvements in glucose transport across the muscle cell membrane. Thus, the effect of exercise training is to reduce blood glucose concentrations and reduce insulin secretion. These responses or adaptations to exercise training will decrease the propensity for type II Diabetes. Additionally, regular physical exercise decreases insulin concentrations in response to carbohydrate administration, which will decrease the propensity for body fat deposition based on the carbohydrate-insulin model of obesity. It must be stated, for insulin to have this profound effect, as stated in the Carbohydrate-Insulin Model of Obesity, individuals must be in positive energy (caloric) balance as stated by the 1st Law of Thermodynamics (energy can neither be created or destroyed but only changes forms); unless hormones such as insulin alter the 1st Law of Thermodynamics. Thus, the effect of exercise training must be taken into consideration in any discussion of glycemic index of carbohydrate containing foods and in reducing the rise in blood glucose due to a large carbohydrate load and of the potential impact of the glucose-insulin model of obesity in body fat deposition. Additionally, the importance of physical exercise in prevention of insulin resistance through muscle glycogen degradation and/or Glut-4 translocation cannot be underestimated. As a result, physical exercise should be a part of each individual’s weekly regimen as it reduces blood glucose and insulin secretion.
